# Whole-exome sequencing identifies novel candidate predisposition genes for familial polycythemia vera

**DOI:** 10.1186/s40246-017-0102-x

**Published:** 2017-04-20

**Authors:** Elina A. M. Hirvonen, Esa Pitkänen, Kari Hemminki, Lauri A. Aaltonen, Outi Kilpivaara

**Affiliations:** 1 Genome-Scale Biology Research Program, Research Programs Unit and Department of Medical and Clinical Genetics, Medicum, University of Helsinki, P.O. Box 63, 00014 Helsinki, Finland; 2 Division of Molecular Genetic Epidemiology, German Cancer Research Center (DKFZ), Heidelberg, Germany; 3 Department of Biosciences and Nutrition, Karolinska Institutet, SE-17177 Stockholm, Sweden

**Keywords:** Myeloproliferative neoplasm, Polycythemia vera, Genetics, Exome sequencing, Familial predisposition

## Abstract

**Background:**

Polycythemia vera (PV), characterized by massive production of erythrocytes, is one of the myeloproliferative neoplasms. Most patients carry a somatic gain-of-function mutation in *JAK2*, c.1849G > T (p.Val617Phe), leading to constitutive activation of JAK-STAT signaling pathway. Familial clustering is also observed occasionally, but high-penetrance predisposition genes to PV have remained unidentified.

**Results:**

We studied the predisposition to PV by exome sequencing (three cases) in a Finnish PV family with four patients. The 12 shared variants (maximum allowed minor allele frequency <0.001 in Finnish population in ExAC database) predicted damaging in silico and absent in an additional control set of over 500 Finns were further validated by Sanger sequencing in a fourth affected family member. Three novel predisposition candidate variants were identified: c.1254C > G (p.Phe418Leu) in *ZXDC*, c.1931C > G (p.Pro644Arg) in *ATN1*, and c.701G > A (p.Arg234Gln) in *LRRC3*. We also observed a rare, predicted benign germline variant c.2912C > G (p.Ala971Gly) in *BCORL1* in all four patients. Somatic mutations in *BCORL1* have been reported in myeloid malignancies. We further screened the variants in eight PV patients in six other Finnish families, but no other carriers were found.

**Conclusions:**

Exome sequencing provides a powerful tool for the identification of novel variants, and understanding the familial predisposition of diseases. This is the first report on Finnish familial PV cases, and we identified three novel candidate variants that may predispose to the disease.

**Electronic supplementary material:**

The online version of this article (doi:10.1186/s40246-017-0102-x) contains supplementary material, which is available to authorized users.

## Background

Myeloproliferative neoplasms (MPNs) are a group of hematological malignancies with enhanced proliferation of myeloid cells due to an acquired mutation of a single hematopoietic stem cell (HSC) resulting in clonal progeny [1, 2]. Expansion of red blood cells in the peripheral blood and bone marrow is the hallmark of polycythemia vera (PV). PV is a chronic, Philadelphia chromosome-negative myeloproliferative disorder with symptoms including fatigue, pruritus, and splenomegaly. The patients have an increased risk of thrombosis, and the disease may further progress to secondary acute myeloid leukemia (sAML) or myelofibrosis [3].

The global annual incidence rate of PV is approximately 1 per 100,000 persons [4]. Most PV patients (approximately 95%) carry a somatic mutation in exon 14 of a non-receptor tyrosine kinase-coding gene Janus kinase 2 (*JAK2*), c.1849G > T (p.Val617Phe, subsequently referred to as *JAK2V617F*) [5–8]. The major diagnostic criteria for PV according to World Health Organization (2008) are exceptionally high hemoglobin and presence of *JAK2V617F* mutation. Other criteria include consistent bone marrow morphology and low erythropoietin (Epo) levels [9, 10]. When diagnosed with PV, most patients are older than 60 years old, and survival depends on the complications and severity of the disease. In younger patients, life expectancy is reduced when compared to the general population [11].

Janus kinase 2 (JAK2) plays a crucial role in the function and maintenance of HSCs [12], as well as in myelopoiesis through binding to various cytokine receptors including erythropoietin receptor (Epo-R), thus, contributing to formation of red blood cells [13]. V617F mutation, which is located within the pseudokinase domain of JAK2 protein, leads to constitutive activity that promotes cytokine hypersensitivity and abnormal signaling through Janus kinase-signal transducer and activator of transcription factor (JAK-STAT) pathway [5–8]. In addition, approximately 3% of PV patients, being *JAK2V617F*-negative, harbor mutations in exon 12 of *JAK2.* The mutations in both exons 14 and 12 induce cytokine-independent proliferation of cells expressing Epo-receptors [14]. Also, certain germline mutations in *JAK2* are predicted to represent a mechanism possibly preceding the acquisition of *JAK2V617F* mutation in PV [15].

Although most PV cases appear to be sporadic, familial clustering is also observed in a subset of cases [16–18]. *JAK2V617F*-positive MPNs are strongly associated with *JAK2* 46/1, or ‘GGCC’ haplotype (rs10974944), which is a common, moderate penetrance predisposition allele [19–22]. Also, a single nucleotide polymorphism (SNP) in the telomerase reverse transcriptase gene (*TERT*), rs2736100, has emerged as another predisposing factor to MPNs [23]. In addition, it has been shown that germline duplication of *ATG2B* and *GSKIP* genes predisposes to familial myeloid malignancies [24], and germline *RBBP6* mutations have been associated with familial MPNs [25]. It is likely that additional inherited genetic factors contribute to PV development as well, since familial clustering of PV suggests the presence of additional susceptibility alleles. High-penetrance predisposition genes to PV have, however, remained unidentified.

Here, we report a Finnish family with four PV patients in two generations (Additional file 1: Figure S1). A family with this many cases is quite exceptional, since PV is a rare disease and usually sporadic. Also, in a familial PV case like here, the age at diagnosis is usually lower, which is an additional indication of the presence of predisposing factors. DNA from all the PV patients in this family was available. The aim of this study was to identify new predisposing gene variants by exome sequencing the DNA of three of the affected family members; the index case and two affected family members (germline) and peripheral blood DNA of the index case. Exome sequencing was not feasible in the fourth PV patient due to very low amount of archived DNA available. The observed, predicted as damaging, germline variants were analyzed in the fourth PV patient of the family, and the variants shared by all four individuals were further screened in six other families with two PV cases in each.

## Methods

### Patient samples

We investigated a Finnish family with four PV patients (Additional file 1: Figure S1). The index case (1.1) was diagnosed with PV at the age of 36, and with myelofibrosis later at the age of 47. The father (1.2) of the index patient was diagnosed with PV at the age of 48, and the aunt (1.9) with PV and acute leukemia at the age of 91. The uncle (1.10) of the index patient was diagnosed with PV at the age of 83. Two individuals in the family had lymphoma; 1.19 was diagnosed with nodular lymphocyte-predominant Hodgkin lymphoma (NLPHL) at the age of 55, and with diffuse large cell non-Hodgkin lymphoma (NHL) later at the age of 68; 1.6 was diagnosed with differentiated diffuse lymphocytic lymphoma at the age of 89. The healthy daughter of the index case is currently 31 years old.

Both peripheral blood and buccal swab samples were available from the index case. Only formalin-fixed paraffin embedded (FFPE) blocks were available from three other family members diagnosed with PV: 1.2, 1.9, and 1.10. Germline DNA was also available from one of two lymphoma patients (1.19) of the family. The second sample set consisted of FFPE blocks from six other Finnish PV families with two first-degree relative cases in five, and two more distant relatives in the sixth family. The study was approved by the appropriate Ethics Review Committee. Samples were derived either after a signed informed consent or after authorization from the National Supervisory Authority for Welfare and Health. The study was conducted in accordance with the Declaration of Helsinki.

### Exome capture and sequencing

DNA was extracted from FFPE blocks with a standard phenol-chloroform method, and the buccal swab sample was extracted with QIAmp DNA Mini kit (Qiagen, Hilden, Germany). DNA from the blood sample was extracted with a standard non-enzymatic TKM buffer-proteinase K method. Sample libraries of gDNA were prepared using NEBNext DNA Library Prep Reagent Set for Illumina (New England Biolabs Ltd. Catalog # E6000), and exomic regions were enriched using Agilent Sure SelectXT Human All Exon V4 + UTRs 50Mb kit (Agilent Technologies, Santa Clara, CA, USA). Paired-end short read sequencing was performed with HiSeq 2000 (Illumina Inc., San Diego, CA, USA) at Karolinska Institutet, Sweden. The DNA library for whole-genome sequencing was prepared with KAPA Hyper Prep Kit (KAPA Biosystems, Wilmington, MA, USA). Paired-end short read sequencing was performed with HiSeq 4000 (Illumina Inc.) at Karolinska Institutet.

### Variant analysis

Exome and genome sequencing data was mapped against the human reference genome GRCh37 (1000 Genomes Project reference hs37d5) with Burrow-Wheeler Aligner (BWA MEM, v.0.7.12, https://arxiv.org/abs/1303.3997), and mapped reads refined following the Genome Analysis Toolkit (GATK) Best Practices workflow including PCR duplicate removal, indel realignment, and base quality score recalibration [26]. Single-nucleotide and short-indel variants were then called using GATK HaplotypeCaller.

The SNV and indel variants in the exome sequencing data were analyzed with an in-house developed analysis and visualization tool (BasePlayer, Katainen et al., manuscript in preparation). A minimum coverage of four reads and the mutated allele present in at least 20% of the reads was required to call a variant. The variants which were present in an in-house control set of 542 Finns [93 whole genome sequenced individuals from the 1000 Genomes Project, 402 whole-genome sequenced individuals from Kuusamo, Finland (Sequencing Initiative Suomi), and 47 uterine leiomyoma patients] were excluded. To exclude common variants, we further filtered the variant set against the Exome Aggregation Consortium (ExAC v.0.3: 3,307 Finns) data [27], setting the maximum allowed minor allele frequency (MAF) in Finnish population to 0.001. Germline variants predicted benign by two independent computational methods PolyPhen-2 [28] and SIFT [29] were excluded.

### Direct sequencing

The shared variants found in the exome data and *BCORL1* cDNA were validated by direct Sanger sequencing. Also, the predisposing germline variant rs10974944 in *JAK2* was checked. DNA was extracted from FFPE blocks with a standard phenol-chloroform method or with NucleoSpin® DNA FFPE XS kit (Macherey-Nagel, Düren, Germany). Primers were designed using Primer3Plus software (http://www.bioinformatics.nl/primer3plus). The DreamTaq™ DNA Polymerase (Thermo Scientific, Waltham, MA, USA) or Invitrogen™, Platinum™, SuperFi™ DNA Polymerase (Thermo Scientific) were used in PCR reactions, and PCR products were purified with A’SAP PCR clean-up method (ArcticZymes, Tromsø, Norway). BigDye Terminator v3.1 sequencing reaction was used in the DNA sequencing, and capillary electrophoresis was performed on an ABI3730xl DNA Analyzer (Applied Biosystems, Foster City, CA, USA) at the Institute for Molecular Medicine Finland (FIMM). The results were analyzed manually using FinchTV v.1.4.0 (Geospiza Inc., Seattle, WA, USA).

For cDNA sequencing, RNA was extracted from index case’s whole blood sample with NucleoSpin® RNA kit (Macherey-Nagel), and reverse-transcripted to cDNA with Promega M-MLV Reverse Transcriptase (Thermo Scientific) according to manufacturers’ protocols.

## Results and discussion

### Novel candidate germline variants for PV predisposition

Here, we have studied a Finnish family with four PV cases with the aim of identifying novel PV-predisposing germline variants. Germline DNA exomes from three affected family members and the peripheral blood DNA exome of the index case were sequenced. The average coverage at each base was 58 reads and 88% of the captured regions had a minimum coverage of four reads. We filtered the called variants based on their predicted, damaging effect on the gene product, and their presence in an in-house control set of 542 Finns. We removed the variants that occurred in any of the 542 Finnish controls (MAF < 0.2%, 95% Cl [0, 0.05%]), leaving us with 12 shared variants; 1 splice site and 11 possibly pathogenic missense variants (Table 1). We then validated the variants by Sanger sequencing in one additional family member diagnosed with PV (1.10).

**Table 1:** A list of damaging (in silico) germline variants in PV patients detected by exome sequencing (three cases)

Gene	Ensembl gene	Ensembl transcript	Genomic location	Variation, cDNA	Variation, protein
ZXDC*	ENSG00000070476	ENST00000389709	Chr3: 126189754	c.1254C > G	p.Phe418Leu
ATN1*	ENSG00000111676	ENST00000356654	Chr12: 7046361	c.1931C > G	p.Pro644Arg
LRRC3*	ENSG00000160233	ENST00000291592	Chr21: 45877228	c.701G > A, rs148872771	p.Arg234Gln
*GNL3*	ENSG00000163938	ENST00000418458	Chr3: 52727477	c.1241A > G	p.Tyr414Cys
*MDC1*	ENSG00000137337	ENST00000376406	Chr6: 30679188	c.2221-1G > T	splice site variant
*ITPR3*	ENSG00000096433	ENST00000374316	Chr6: 33635026	c.1672C > T, rs780906252	p.Arg558Cys
*FAM135A*	ENSG00000082269	ENST00000418814	Chr6: 71190668	c.607G > A, rs143901584	p.Val203Met
*SLC2A12*	ENSG00000146411	ENST00000275230	Chr6: 134312391	c.1756C > T, rs200847615	p.Pro586Ser
*WDR86*	ENSG00000187260	ENST00000334493	Chr7: 151097265	c.226G > A, rs199824863	p.Asp76Asn
*CSMD1*	ENSG00000183117	ENST00000537824	Chr8: 3165238	c.3929C > T	p.Ala1310Val
*SLC24A2*	ENSG00000155886	ENST00000341998	Chr9: 19786283	c.582A > G, rs368590535	p.Ile194Met
*ITPKC*	ENSG00000086544	ENST00000263370	Chr19: 41224132	c.1092C > G, rs143757004	p.Asp364Glu

The three shared variants in all four cases are marked with an *asterisk*. Genome assembly: GRCh37

From these variants predicted as damaging, 1.10 carried three rare single-nucleotide variants (SNVs): c.1254C > G (p.Phe418Leu) in *ZXDC* (ENST00000389709); c.1931C > G (p.Pro644Arg) in *ATN1* (ENST00000356654); and rs148872771, c.701G > A (p.Arg234Gln) in *LRRC3* (ENST00000291592). We screened the three variants in lymphoma patient 1.19, who carried the variant in *LRRC3*. Also, we checked for the three variants in the germlines of eight PV patients from six other Finnish families, but the variants were not observed. It may still be possible, however, that some of the three variants or other variants in these genes may play a role in PV development. We identified also one rare benign (PolyPhen-2, SIFT) missense SNP, rs144332650, c.2912C > G (p.Ala971Gly), and in *BCORL1* (ENST00000540052) in all four PV patients in the family. Mutations in *BCORL1* have been associated with the leukemogenic process in AML [30–32]. PV patients in six other families did not carry the variant.

Zinc-finger X-linked duplicated family member C (ZXDC) belongs to the ZXD family of transcription factors, which has been observed to regulate transcription of major histocompatibility complex (MHC) class I and II genes in antigen presenting cells [33]. In addition to zinc fingers, ZXDC contains a transcriptional activation domain, and a specific domain used for interaction with a transcriptional co-factor class II *trans*-activator (CIITA), which leads to CIITA binding to promoter elements involved in constitutive MHC class II expression [33, 34]. By reducing the expression of ZXDC, CIITA activation of MHC class II gene transcription is significantly reduced [33]. Thus, downregulation of *ZXDC* may contribute to carcinogenesis and malignant progression of tumors by participating in the suppression of MHC class II genes. The mutated site of *ZXDC* identified in our study is the first amino acid of the ninth zinc finger repeat. Zinc fingers are necessary for full activity of cooperation with CIITA [33]. Beyond this role of acting as a co-factor in CIITA function, the *ZXDC* gene function is unknown. ZXDC is enriched in myeloid lineages and has been observed to regulate transcription of key genes during myeloid cell differentiation [35]. During hematopoiesis, it is expressed especially in stem and progenitor cells in the bone marrow, myelocytes, and leukocytes (BloodSpot http://nar.oxfordjournals.org/content/early/2015/10/26/nar.gkv1101.abstract, www.proteinatlas.org). Solely based on gene function, *ZXDC* variant would be the most attractive predisposition candidate of the three, but further studies are warranted.

Atrophin-1 (ATN1) is a nuclear transcriptional corepressor, and aberrant form of ATN1 is associated with neurodegenerative diseases such as dentatorubral-pallidoluysian atrophy (DRPLA), and cancer in humans [36]. It is ubiquitously expressed in neurons [37] and widely in various other tissues, e.g., in hematopoietic cells in the bone marrow (www.proteinatlas.org). ATN1 contains glutamine-repeats, and two arginine-glutamic acid dipeptide-like repeats (RE-repeats) [36]. In neuronal nuclei, ATN1 has been shown to interact with a transcriptional repressor Eight twenty-one (ETO) protein [38]. In normal hematopoiesis, it is widely expressed in differentiated blood cells, but the expression is lower in stem and progenitor cells (BloodSpot). Thus, it is likely that ATN1 interaction with ETO has no contribution to erythropoiesis or development of myeloproliferative diseases. On the other hand, in patients with AML, ATN1 is expressed more substantially (BloodSpot) compared to normal counterparts. Nevertheless, its normal function is not completely understood.

Little is known about the function of Leucine-rich repeat containing 3 (LRRC3) gene product, but it is widely expressed in different tissues, including the bone marrow. Most malignancies display moderate cytoplasmic-positive staining, and the strongest expression is observed in colorectal cancers (www.proteinatlas.org). One lymphoma patient of the family with DNA available carried the rare variant rs148872771 in *LRRC3*, too, which may indicate the particular variant not being responsible for PV predisposition exclusively.

The SNP in *JAK2*, rs10974944, was identified in all the PV patients in the family. Individuals 1.1, 1.2, and 1.10 were homozygous for the risk variant in their germline (GG genotype), whereas 1.9 was heterozygous (CG genotype). Also, all eight PV patients from the six other families carried the risk variant: two of them were heterozygous, and six were homozygous. The SNP in *TERT*, rs2736100, was also checked from the patients. All the PV patients in the studied family were homozygous for the risk variant (CC).

Germline duplication of *ATG2B* and *GSKIP* has been shown to predispose to familial myeloid malignancies [24]. A possible duplication was checked for in the whole-genome sequence data of the index case by visualizing the depth of coverage in the region. The duplication had not occurred in the index patient’s genome.

### Detection of somatic variants

We identified the most frequent somatic variation in PV patients, *JAK2V617F*, in index case’s blood sample. Loss of heterozygosity (LOH) in the 12 damaging gene variants detected by exome sequencing was looked for in the index case’s blood sample, but only the variants c.582A > G (p.Ile194Met) in *SLC24A2* and c.3929C > T (p.Ala1310Val) in *CSMD1*, in addition to *JAK2V617F*, showed clear LOH. In known MPN-associated genes, the index case carried two missense variants, c.680C > T (p.Thr227Met) in *FLT3* and c.5162 T > G (p.Leu1721Trp) in *TET2* (Illumina TruSight® Myeloid Sequencing Panel), predicted as possibly damaging by PolyPhen-2 and SIFT. In addition, we identified possibly damaging missense variants c.3263C > T (p.Ser1088Phe) and c.1235C > T (p.Ala412Val) in *FANCA*, which is one of the genes associated with other myeloid malignancies [39, 40].

*BCORL1* gene is located on the X chromosome. The index case being a woman, we checked which of the alleles was expressed; the *BCORL1* variant or the wild-type. By Sanger sequencing the cDNA, we identified the expression of *BCORL1* variant.

## Conclusions

Identification of predisposing genes and mutations is important for families with PV susceptibility and acknowledging family history is essential in order to unveil the genetic background. New hereditary gene defects may lead to screening and genetic counseling of family members and may improve the diagnosis and treatment thus affecting the quality of life. Also, the identification of specific gene mutations gives the possibility to screen individuals at higher risk. This is the first report on Finnish familial PV cases, and we identified three candidate predisposition variants by exome-sequencing. We would like to present these genes as candidates for PV susceptibility and for further validation by the research community.

## Additional file

Additional file 1:Figure S1. Pedigree of the Finnish family with four cases of polycythemia vera, and two cases of lymphoma. Exome-sequenced family members are marked with an *asterisk* and the individual used for validation with a *small square*. The index case was also whole-genome sequenced (peripheral blood DNA), in addition to germline exome sequencing. Acute leukemia is marked with a *small circle*. The pedigree has been slightly modified for confidentiality. (PDF 1382 kb)
