# Supplementary figures and images for: Whole-exome sequencing identifies novel candidate predisposition genes for familial polycythemia vera
Source: Hum Genomics. 2017 Apr 20;11:6. doi: 10.1186/s40246-017-0102-x (PMC5397753; doi:10.1186/s40246-017-0102-x)

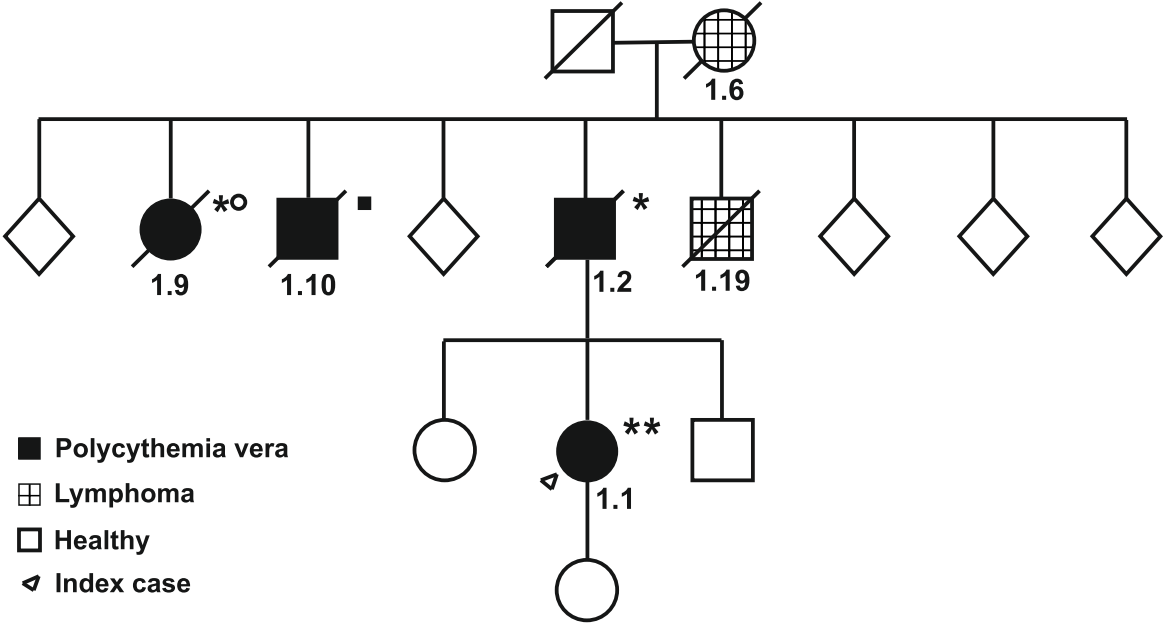

Supplement: Additional file 1:Figure S1. — Pedigree of the Finnish family with four cases of polycythemia vera, and two cases of lymphoma. Exome-sequenced family members are marked with an asterisk and the individual used for validation with a small square. The index case was also whole-genome sequenced (peripheral blood DNA), in addition to germline exome sequencing. Acute leukemia is marked with a small circle. The pedigree has been slightly modified for confidentiality. (PDF 1382 kb) [file 40246_2017_102_MOESM1_ESM.pdf]
